# Bardet-Biedl Syndrome, Crohn Disease, Primary Sclerosing Cholangitis, and Autoantibody Positive Thyroiditis: A Case Report and A Review of a Cohort of BBS Patients

**DOI:** 10.1155/2012/209827

**Published:** 2012-08-15

**Authors:** Ugur Halac, Denise Herzog

**Affiliations:** Division of Gastroenterology, Hepatology and Nutrition, Department of Pediatrics, Hospital Sainte-Justine, Université de Montréal, Québec, Canada H3T 1C5

## Abstract

Bardet-Biedel syndrome (BBS) is a rare autosomal recessive, genetically heterogeneous ciliopathy. Although the disease has been described in a patient with psoriasis, individuals with BBS are not known to be at risk of developing autoimmune disorders. Our objective was to describe a 14-year-old patient with BBS who presented with Crohn disease (CD), primary sclerosing cholangitis (PSC), and thyroiditis in the context of a cohort review at Sainte-Justine Hospital and to alert clinicians to the increased risk of autoimmune disorders in these patients. The cohort contained fifteen patients (9 boys), followed from 1968 to 2009 during a median period of 12 years (range 9 months–26 years). Three of the 15 patients (20%) developed a chronic autoimmune disease: one had juvenile rheumatoid arthritis; a second one had type 1 diabetes mellitus in association with Hashimoto thyroiditis and psoriasis; a third one developed CD, PSC, and Hashimoto thyroiditis. As chronic autoimmune diseases occurred in 20% of our cohort of children with BBS, it is appropriate to keep this association in mind during the followup.

## 1. Introduction 

Bardet-Biedel syndrome (BBS) is an autosomal recessive disorder, characterized by the following major criteria: early rod-cone dystrophy, polydactyly, obesity, hypogonadism, mental retardation, and renal abnormalities (including parenchymal cysts, calyceal clubbing, foetal lobulation, multicystic dysplastic kidneys, unilateral agenesis, renal calculi, vesicoureteric reflux, bladder obstruction, hydronephrosis, horseshoe kidney, and ectopic kidney) [1]. Minor criteria include cardiopathy, hepatic fibrosis, type II diabetes mellitus, short stature, neuropsychiatric problems (speech or expressivity troubles, slow ideation, and cognitive troubles), dental abnormalities, and pigmented nevi. The clinical diagnosis is based on the existence of 4 major, or 3 major plus 2 minor features [2]. 

To date, BBS is genetically heterogeneous with 14 BBS genes responsible for 14 identified phenotypes [3–5]. The pathophysiology is not completely understood but recent studies confirmed the crucial role of proteins encoded by the BBS genes as components of the centrosome and/or basal body, and their implication either in the constitution of the primary cilia or in their transport [6]. For this reason, BBS is currently considered as a “ciliopathy” [7], and the protein complex responsible for vesicular trafficking of membrane proteins to the primary cilium is called *BBsome* [8]. Recent discoveries indicated that ciliary dysfunction was associated with human obesity. It has been first demonstrated that obesity in BBS mice was associated with hyperleptinemia and leptin resistance [9, 10]. Subsequently, it has been reported that BBS proteins are required for leptin receptor (LepR) signaling using a BBS knockout mouse models [10, 11] which reproduce the major features of the human phenotype including obesity. 

Some case reports describe inflammatory conditions (such as psoriasis) in BBS patients but no patient with BBS and chronic autoimmune disorders, such as inflammatory bowel disease (IBD), primary sclerosing cholangitis (PSC), and Hashimoto thyroiditis (HT) has been reported to date. Herein, we report the case of an 14-year-old boy with BBS10 who developed Crohn disease (CD), PSC, and HT, and briefly review a cohort of 15 patients with BBS followed in our institution to assess if these patients with BBS are at increased risk of developing chronic autoimmune diseases. 

## 2. Material and Methods

A single centre, retrospective chart review of patients followed at Sainte-Justine Hospital from 1969 to 2007 was conducted after approval by the institutional Research Ethics Board. Charts were reviewed and following data were recorded: age, gender, date of birth, age at diagnosis, presenting criteria, results of genetic tests, duration of followup, presence of any chronic inflammatory, or autoimmune diseases. Appropriate descriptive statistics were used for the analysis of the cohort's clinical features.

## 3. Results and Case Presentation

### 3.1. BBS Cohort

The charts of fifteen patients (9 boys), born between 1968 and 2002, were studied. The median followup was 12 years (range 9 months–26 years). Every patient was diagnosed as BBS based on severe obesity and at least 3 other major criteria, and results of genetic testing were available in 2 children. 

During the followup, two children were transplanted for kidney failure at 14 and 16 years of age due to a BBS associated nephropathy. These patients developed progressive renal insufficiency secondary to multicystic dysplastic kidneys. No evidence of autoimmune nephropathy was observed in both children. Two patients died because of renal insufficiency: the first one on the waiting list for transplantation and the second one after renal transplantation due to surgical complications and vascular thrombosis. Currently, three children are still followed in our centre, while 10 patients have been transferred to adult care. Clinical characteristics of this cohort are reported in Table 1.

Three patients developed inflammatory disorders (Table 1). The first one had juvenile rheumatoid arthritis, without uveitis, early in life but data were limited concerning the age of onset. The second child developed type 1 diabetes mellitus at 7 years of age and was treated with insulin. This diagnosis was made based on the age of this child but without any specific biomarkers and/or genetical screening. Antithryroid peroxydase (anti-TPO) positive HT (autoantibodies between 1600 and 25600, normal 0–34) were found at the age of 13. During his 15th year, he also presented with lesions of psoriasis requiring topical treatment with corticosteroids. The third member of this cohort with CD, PSC, and HT is described further below. 

### 3.2. Case Report

**Figure 1:**
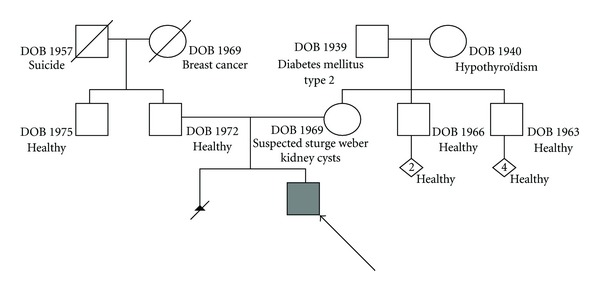
Familial pedigree of the reported patient. The patient has nonconsanguineous French Canadian parents without history of inflammatory bowel disease. First-degree relatives are healthy except the mother who presents with renal cysts, possible Sturge-Weber syndrome, and previous abortion secondary to foetal anencephaly and abnormal thorax.

This patient, born in 1997, is the only child of nonconsanguineous French Canadian parents. There is no familial history of chronic autoimmune disease. The father is healthy and tall (198 cm, +1.5 standard deviation) and the mother has a huge naevus flammeus of the left facies and partial deafness suggesting Sturge-Weber syndrome but was not investigated. At the age of 29 years, a first pregnancy required a therapeutic abortion for foetal anencephaly and an abnormal thorax as well as renal cysts. The family pedigree is presented in Figure 1.

At the age of 40, after an uneventful pregnancy with prenatal diagnosis of foetal bilateral renal polycystic disease, the mother had an uneventful delivery. The full-term male newborn, weighing 3.2 kg, presented with superior and inferior postaxial polydactyly, an incomplete foreskin, a naevus flammeus on the neck, a flattened angioma on the philtrum and on the nose, and a mild erythema on the left eyelid. The renal US confirmed the prenatal diagnosis revealing bilaterally increased kidneys (57–60 mm of longitudinal length) with hyperechogenic and ill differentiated cortical and medullar areas, and the presence of several cysts. 

Early in life, the child presented a developmental delay: crawling at 18 months, sitting at 24 months, speech acquisition after 3 1/2 years. The body mass index (BMI) was also abnormally increased early in life (31 and 32 at 2 1/2 and 5 years old, resp.). In 2006, at 9 years of age, the suspected diagnosis of BBS was confirmed (homozygous *c.274insT* (*p.C91fsX95*) variation in the *BBS10* gene). At the age of 10, normal creatinine clearance (102 mL/min/1.73 m^2^), normal blood pressure, a decreased visual acuity and night vision without signs of retinal dystrophy, and right sided mild deafness and cholesteatoma of the right ear were documented. During this period, BMI continued to increase and reached 36. At the end of his 10th year, the patient consulted for 5-6 liquid, nonbloody bowel movements increasingly present over the previous 7 months, and a weight loss of 13 kg. He had no abdominal pain and physical examination of the abdomen was normal. Serum albumin was 23 g/L (normal = 41–54), hemoglobin 102 g/L (normal = 115–155), serum iron 2.7 umol/L (normal = 11.6–31.3), and the sedimentation rate was 44 mm/h. Liver function tests were normal at this period. Abdominal sonography showed a thickened wall of the terminal ileum, the ascending and the descending colon. Upper and lower digestive endoscopies revealed deep ileal ulcerations, normal colonic mucosa, and mild nonspecific gastritis. The diagnosis of CD was confirmed by the presence of ileal ulcerations and granulomas, oedema and hypercellularity of the colonic mucosa as well as by a dense chronic inflammatory infiltrate of the rectum. No extraintestinal signs of CD were observed. Remission was achieved with corticosteroids and is maintained with oral 6-mercaptopurine (1 mg/kg/day). He has also regained his former weight (BMI of 39.5). Despite morbid obesity, he does not present any criteria of metabolic syndrome: serum insulin, glucose, and triacylglycerols levels are normal, as well as his blood pressure. At the time the diagnosis of CD was made, the patient also had low serum TSH level (0.05 mU/L, normal = 0.1–6.5), elevated free T4 concentration (31.5 mmol/L, normal = 7.5–17.2), and presence of anti-TPO (45, normal = 0–34), compatible with an autoimmune thyroïditis. Under immunosupprression, TSH/free T4 status and anti-TPO antibodies levels normalized. Serum level of immunoglogulins (Ig) A, G, and M was normal. The patient has also been shown to be HLA DQ2 antigen negative and DQ8 antigen positive. 

Recently, this adolescent presented with persistently increased levels of serum aminotransferases: alanine aminotransferase (ALT) and aspartate aminotransferase (AST) at 101 and 67, respectively, and gamma-glutamyl transpeptidase. (GGT) at 59 U/L during a 6-months period without any clinical sign or complaint. In our hospital, the upper limit for normal ALT, AST, and GGT is 25–34 U/L, 43–70 U/L, and 3–43 U/L, resp.). Etiologies of hepatitis were systematically explored. In particular, we thoroughly ruled out usual infectious causes (Hepatitis A, B, C, and D viruses, Epstein-Barr virus, cytomegalovirus, adenovirus, herpes group viruses, parvovirus, and echoviruses), Wilson disease, and autoimmune hepatitis. Toxic causes related to 6-mercaptopurine treatment were also excluded by weaning him off the treatment for a prolonged time. A magnetic resonance cholangiography was performed and revealed features compatible with PSC, in particular peripheral areas of high T2-weighted signal intensity in the liver parenchyma, intrahepatic bile ducts strictures, and secondary dilatation. In addition, peri-portal edema and peri-portal inflammation have also been observed as regions of low signal intensity on T1-weighted images and as intermediate signal between liver and bile on T2-weighted images. These abnormalities were highly suggestive of a diagnosis of PSC. A liver biopsy was proposed but parents declined this option. The patient has been treated with ursodeoxycholic acid treatment (500 mg twice a day) and serum aminotransferases have normalized. Currently, he is free of any symptoms or signs of chronic hepatopathy. 

## 4. Discussion 

Development of 3 autoimmune diseases over a 14-year period in one patient led us to ask whether other BBS patients were at risk of developing chronic inflammatory and autoimmune disorders, especially IBD, and to review the cohort of BBS patients in our hospital.

BBS is estimated to occur in 1/125.000 to 160.000 inhabitants of European countries and in 1/13.500 to 1/17.500 people of Middle East countries and Newfoundland [12]. The spectrum of the clinical features is large because of the affection's phenotypic variability with intrafamilial as well as interfamilial disparity. This disorder appears, at least by some gene mutations, as being in the frontier between classical monogenic Mendelan heredity and multifactorial heredity [13]. A new model of triallelic heredity is current since the pioneer work of Katsanis et al. who reported that in some families, heterogeneous mutations can influence the penetrance and the expressivity of the phenotypes [14]. Hence, the latter can be extremely different when mutations on a second BBS gene exist. For this reason, BBS became the first example of triallelic inheritance described in humans.

The genetic defect found in the reported patient is a frameshift mutation on chromosome 12q21.2, described in almost 50% of patients with *BBS 10* [15]. The *BBS 10* gene, incriminated in almost 20% of BBS cases, encodes a vertebrate-specific chaperoninlike protein [15]. As member of the chaperonines type II, a family of multistructured proteins, *BBS10* is implicated in the folding and spatial conformation of target proteins [4], and in the BBsome assembly [8], a protein complex responsible for the vesicular trafficking of membrane proteins to the primary cilium. The possible relationship between this genetic defect and chronic inflammatory or autoimmune diseases reported in BBS patients is not established. But, 20% of patients with BBS in our cohort presented with chronic inflammatory or autoimmune disorders. Admittedly, this observation should be first confirmed in larger cohorts of patients with BBS or congenital ciliopathy. However, some observations are relevant. It has been demonstrated that the adipose tissue of some obese individuals produces higher levels of the proinflammatory cytokine TNF and other proinflammatory factors [16]. Obesity in BBS seems more specific, and pathophysiologicial theories are recent. Studies clearly suggest that ciliary dysfunction is associated with human obesity, despite the fact that adipocytes are not considered as a ciliated cell (http://www.bowserlab.org/primarycilia/cilialist.html). First, it has been demonstrated that obesity in BBS mice was associated with hyperleptinemia and LepR [9, 10]. Subsequently, it has been found that BBS proteins were required for LepR signaling in the hypothalamus, by using a BBS knockout mouse model [10, 11] which reproduce the major features of the human phenotype, including obesity. These data suggest that BBS genes mediate LepR trafficking and that impaired LepR signaling induces energy imbalance in BBS [10]. A defect in *BBS10* gene impairing the ciliogenesis and activating proadipogenic pathways was also evidenced [17]. Therefore, it seems that the pathogenesis of BBS-associated obesity by a defect of ciliary cells is unique in BBS patients. Are the adipocytes of these patients different, and do they have precise abilities in order to induce specific impairment in specific conditions? Further studies are necessary to assess this hypothesis and to explore accurately the relationship between BBS and autoimmune disorders. In the case of reported patient with CD, PSC, and HT, the morbid obesity without any hyperlipidemia could be due to central leptin resistance. However, this boy also carries a HLA *DQ8* antigen, an antigen commonly linked to autoimmune diseases in the human population. 

In conclusion, we described a patient with BBS, ileal CD without family history of IBD, PSC, and Hashimoto thyroiditis, and found a 20% prevalence of chronic inflammatory or autoimmune diseases in our cohort of patients with BBS. These observations should encourage physicians to explore carefully and rule out chronic inflammatory or autoimmune disorders, including IBD, in the followup of BBS patients.

## Figures and Tables

**Table 1:** Clinical characteristics of a cohort of 15 patients with Bardet-Biedl syndrome.

Patients	Date of birth	Gender	Molecular testing of BBS gene defect (age at the test (years))	Follow-up period duration (years)	Associated chronic inflammatory or auto-immune disorders
1	8/03/1968	F	No	26	/
2	10/09/1970	M	No	17	/
5	9/10/1970	F	No	8.2	/
3	05/11/1973	M	No	21.5	/
4	9/07/1974	F	No	17	/
6	10/12/1977	F	No	16.1	/
7	11/01/1979	F	No	14.2	/
8	30/06/1980	F	No	15.3	/
9	8/12/1983	M	No	9.3	/
10	5/04/1984	M	No	8.1	Juvenile rheumatoid arthritis
11	16/06/1985	M	No	17.9	Type 1 diabete mellitus, Hashimoto thyroiditis, Psoriasis
12	22/04/1986	M	No	0.8	/
13	30/12/1988	M	Yes (14)	15.2	/
14	04/11/1997	M	Yes (9)	14	Crohn's disease, primary sclerosing cholangitis, Hashimoto thyroiditis
15	04/09/2002	M	No	9.3	/

F: female, M: male.
